# Hybrid LiDAR–radar at 9 μm wavelength with unipolar quantum optoelectronic devices

**DOI:** 10.1515/nanoph-2025-0265

**Published:** 2025-09-01

**Authors:** Livia Del Balzo, Djamal Gacemi, Jihye Baik, Bruno Martin, Axel Evirgen, Grégoire Beaudoin, Konstantinos Pantzas, Isabelle Sagnes, Angela Vasanelli, Carlo Sirtori

**Affiliations:** Laboratoire de Physique de l’École normale supérieure, ENS, Université PSL, CNRS, Sorbonne Université, Université Paris Cité, 75005, Paris, France; Thales Research and Technology, Palaiseau, France; III-V Lab, Palaiseau, 91120, France; Centre de Nanosciences et de Nanotechnologies, Université Paris-Saclay, CNRS, Palaiseau, 91120, France; Laboratoire de Physique de l’ENS, Université Paris Cité, Ecole normale supérieure, Université PSL, Sorbonne Université, CNRS, 75005, Paris, France

**Keywords:** quantum cascade laser, unipolar device, mid-infrared, LiDAR, Stark modulator, quantum well infrared photodetector

## Abstract

Unipolar quantum optoelectronics is emerging as a promising semiconductor platform for developing mid-infrared applications, particularly spectroscopy and free-space communications. In this work, we present a proof of principle of a unipolar quantum optoelectronic hybrid Lidar – Radar for the measurement of the position and speed of a moving target. The system operates at 9 µm wavelength and is composed of a quantum cascade laser, a Stark modulator and a metamaterial quantum well infrared photodetector. The laser amplitude is modulated with a chirped radio-frequency subcarrier, and the backscattered light is detected coherently on the metamaterial quadratic receiver. With our system we have measured a ranging distance of 150 cm with an intrinsic resolution of 15 cm. Our demonstration is a first milestone towards combined communication and ranging systems in a transparency window of the atmosphere.

## 1 Introduction

The invention of quantum cascade lasers [1] in 1994 has fostered the realization of several unipolar quantum optoelectronic devices [2], [3], [4], based on intraband transitions in semiconductor quantum wells. Thanks to the continuous improvement of their performances, unipolar quantum optoelectronics is increasingly gaining importance for several applications in the mid-infrared wavelength range, in particular spectroscopy and free-space communications [4], [5], [6], [7]. Unipolar quantum optoelectronics presents indeed several peculiarities and advantages. First of all, unipolar devices are realized by using materials and fabrication processing already developed for telecom optoelectronics, and thus fully exploit its technological maturity. These devices are also intrinsically fast [8], [9], due to their extremely short excited-state lifetime, in the order of 1 ps. [10]. Furthermore, wavelength agility is one of their major characteristics: the operation wavelength of the devices is not set by the energy gap of the material, but rather by the electronic potential designed in the semiconductor heterostructures, that can be engineered at will. This allows the realization of semiconductor optoelectronic devices operating in the transparency windows of the atmosphere, between 4–5 µm and 8–14 µm, which is extremely appealing for free-space optics [11] applications and spectroscopy [12].

In this context, unipolar quantum optoelectronics has a great potential for the realization of LiDARs, i.e. systems capable of detecting light reflected from a target in order to measure its distance and speed. In LiDARs, optical ranging can be performed using different waveforms (pulsed or amplitude-/ frequency-modulated continuous wave), while detection can be either direct or coherent, i.e. in a heterodyne configuration. Unipolar quantum optoelectronics provides today all the necessary building blocks to realize LiDAR systems operating in the mid-infrared wavelength range. Indeed, quantum cascade lasers are powerful and compact mid-infrared sources, that can be directly modulated [6]. Unipolar detectors are excellent coherent receivers [13], thanks to their large bandwidth and high saturation power. Unipolar modulators have recently demonstrated to be very efficient for high speed data transmission in the free-space [5], and can be used to modulate the amplitude or the phase [14], [15] of the beam emitted by a continuous wave QCL. Despite the existence of all these building blocks, up to now mid-infrared LiDARs are mostly based on parametric oscillators or CO_2_ lasers. To the best of our knowledge, there is only one demonstration of a mid-infrared LiDAR employing a frequency modulated quantum cascade laser [16]. Recently, a mid-infrared laser chaos LiDAR was also developed, based on an interband cascade laser [17].

In this work, we experimentally demonstrate a novel detection and ranging system, a hybrid LiDAR – radar, based on unipolar quantum optoelectronic devices operating at 9 µm. Our experiment is inspired by frequency-modulated continuous wave (FMCW) detection and ranging, one of the most common techniques used in microwave radar and LiDAR systems. FMCW is usually employed to realize LiDARs either by directly modulating the frequency of the optical carrier or by modulating the intensity with a radiofrequency (RF) subcarrier [18]. Our system is based on a different concept. The continuous wave laser is amplitude modulated with a radio-frequency subcarrier, which itself is linearly frequency-modulated [19] by an external Stark modulator. The backscattered light is detected coherently, by mixing it with a reference optical signal on a unipolar coherent receiver. Our system is, therefore, a hybrid LiDAR – radar because both the RF subcarrier and the mid-infrared carrier are exploited for distance and speed measurements, respectively. Indeed, the RF subcarrier is utilised to extract the distance, like in a *FMCW Radar*, while the mid-infrared carrier measures the velocity of the moving target thanks to the Doppler effect, like in a *Li* *DAR*. In our system the integration time for the measure of speed and distance can be set independently. In particular, the velocity resolution can be adjusted by setting an integration time regardless the frequency chirp used for the distance measurement. A similar concept was developed by using a carrier in the telecom range [20], [21].

The paper is organised as follows. We first introduce the principle of operation of our hybrid LiDAR–radar system, and the detection techniques for the measurement of the distance and velocity of a moving target. In Section 3, we present the Stark modulator, its characterization and the demonstration of a crucial property for LiDAR applications, i.e. its FM linearity. The results on the measurement of distance and velocity of a moving mirror target are presented in Section 4. Finally, we summarize the results and present the perspectives of this work.

## 2 Principle of the hybrid LiDAR – Radar system

**Figure 1:**
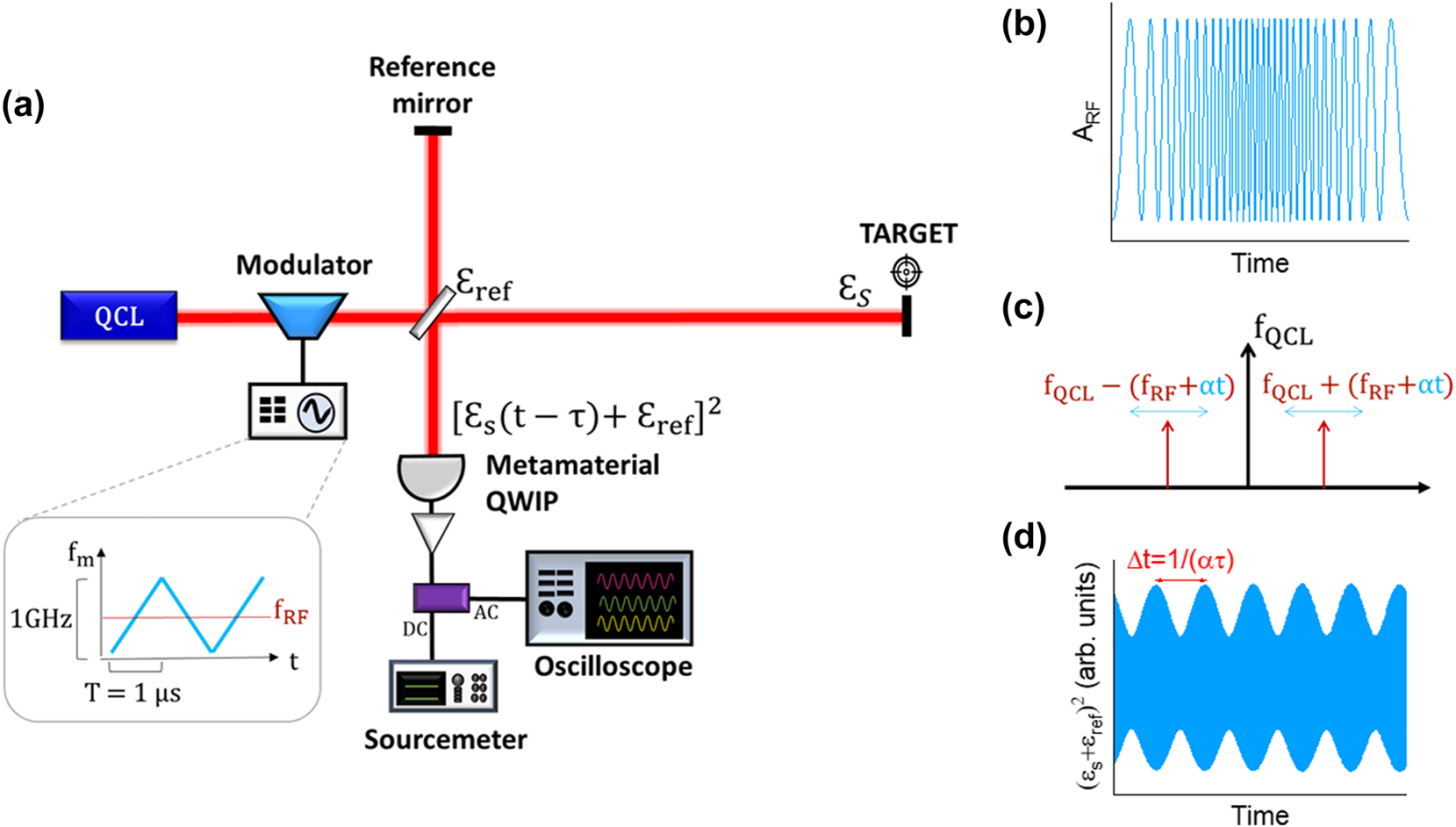
Principle of operation of the hybrid LiDAR-radar. (a) Schematic of the hybrid LiDAR–radar setup. The bias applied on the modulator is a sinusoidal waveform whose frequency f_m_ is linearly swept from 100 MHz to 1.1 GHz. The ramp period, T = 1 μs, is the maximum value allowed in these conditions by the arbitrary waveform generator. (b) RF modulated subcarrier given by $A_{RF}\left(t\right)$ . (c) Modulation mechanism in the frequency domain: the Stark modulator driven by the chirped frequency *αt* creates two instantaneous sidebands around the carrier frequency *f*_QCL_. The linear chirp of the RF subcarrier shifts the frequency of the sidebands. (d) Square modulus of the field intensity impinging on the detector.

Our system is sketched in Figure 1a. The mid-infrared carrier is emitted by a distributed feedback quantum cascade laser (QCL) at frequency *f*_QCL_ = 33 THz. The amplitude of the electric field emitted by the laser, *A*_QCL_, is modulated with a linearly chirped frequency by applying a RF signal on an external Stark modulator:

$$\varepsilon_{\text{ref}}=A_{RF}\left(t\right)\cos\left(2\pi f_{\text{QCL}}t\right)$$

$$A_{RF}\left(t\right)=A_{\text{QCL}}-A_{m}\cos\left(2\pi \left(f_{RF}t+\frac{\alpha }{2}t^{2}\right)\right)$$

Here $A_{RF}\left(t\right)$ is the amplitude modulation at chirped frequency (see Figure 1b), *A*_*m*_ is the modulation depth, *f*_*RF*_ is the modulation frequency (i.e. the RF subcarrier) and *α* is the modulation rate, i.e. the slope of the frequency as a function of time, defining the linear chirp. In the frequency domain, linearly chirping the modulation frequency corresponds to sweeping the amplitude modulation sidebands of the optical carrier *f*_QCL_. Note that the optical carrier at frequency *f*_QCL_ is not modulated, as the linear chirp acts on the amplitude and generates instantaneous shifting sidebands at a time *t* (Figure 1c).

The target mirror, initially at fixed distance *d* from the reference mirror, is moved along a delay line to introduce a velocity component, *v*. The back-reflected signal from the target is then mixed with the reference signal, ε_ref_, on a metamaterial quantum well infrared photodetector (QWIP) [13], which operates as a coherent receiver. The electric field of the backscattered signal can be written as:

$$\begin{array}{ll} \varepsilon_{S}\left(t\right) & =\left[A_{\text{QCL}}-A_{m}\cos\left(2\pi \left(f_{RF}+f_{\mathrm{dop}}^{RF}+\frac{\alpha }{2}\left(t-\tau \right)\right)\right.\right.\\ & \;\left.\left.\times \left(t-\tau \right)\right)\right]\cos\left(2\pi \left(f_{\text{QCL}}+f_{\text{dop}}^{\text{QCL}}\right)\left(t-\tau \right)\right) \end{array}$$

In this expression, *τ* is the time delay induced by the distance *d* between the target and the reference mirror, $\tau =\frac{2d}{c}$ , while $f_{\text{dop}}^{RF\left(\text{QCL}\right)}=\frac{2vf_{RF\;\left(\text{QCL}\right)}}{c}$ is the frequency shift induced by the Doppler effect that can be added on *f*_QCL_ or *f*_*RF*_.

Figure 1d presents the square amplitude of the electric field resulting from the beating between the reference and the backscattered signal obtained for a still target ${\left(\varepsilon_{S}\left(t\right)+\varepsilon_{\text{ref}}\right)}^{2}$ , which sets the self-homodyne signal on the detector. The distance is extracted from the envelope of the self-homodyne signal, at frequency Δ*f*: $d=\frac{c\Delta f}{2\alpha }$ , while the velocity is extracted through the Doppler shift of the optical carrier, which is much easier to be detected than the shift of the RF frequency.

## 3 Characterization of the Stark modulator and FM linearity

Our modulator is based on the linear Stark effect in a system of asymmetric tunnel-coupled quantum wells [22]. It consists of 50 repetitions of a semiconductor heterostructure composed of two GaInAs quantum wells, with thicknesses 6.4 nm and 2.8 nm, separated by an AlInAs barrier of 0.85 nm thickness. The larger quantum well is doped with 3 × 10^18^ cm^−3^ Si donors. A 20 nm barrier separates each period so as to prevent tunnel coupling. The structure is inserted between two contact layers n-doped with a density 10^18^ cm^−3^. The heterostructure has been grown by molecular beam epitaxy on semi-insulating InP:Fe.

The principle of operation of the device is the following. An electric field applied on the structure linearly shifts the absorption energy of the intraband transition between the ground and the first excited state of the system of coupled quantum wells, *E*_12_ = *E*_2_ − *E*_1_. When a laser beam impinges on the modulator, the amplitude of the transmitted signal at the laser frequency depends on the intraband absorption, which in turn depends on the applied electric field. Note that light modulation is induced by a bias voltage, modifying the energy position of the absorption peak, without any charge displacement. Consequently, the only physical factor limiting the device speed is the electron excited state lifetime, which is in the order of 1 ps. In practice, the modulator bandwidth is limited by the parasitic capacitance and resistance arising from the device architecture and packaging.

**Figure 2:**
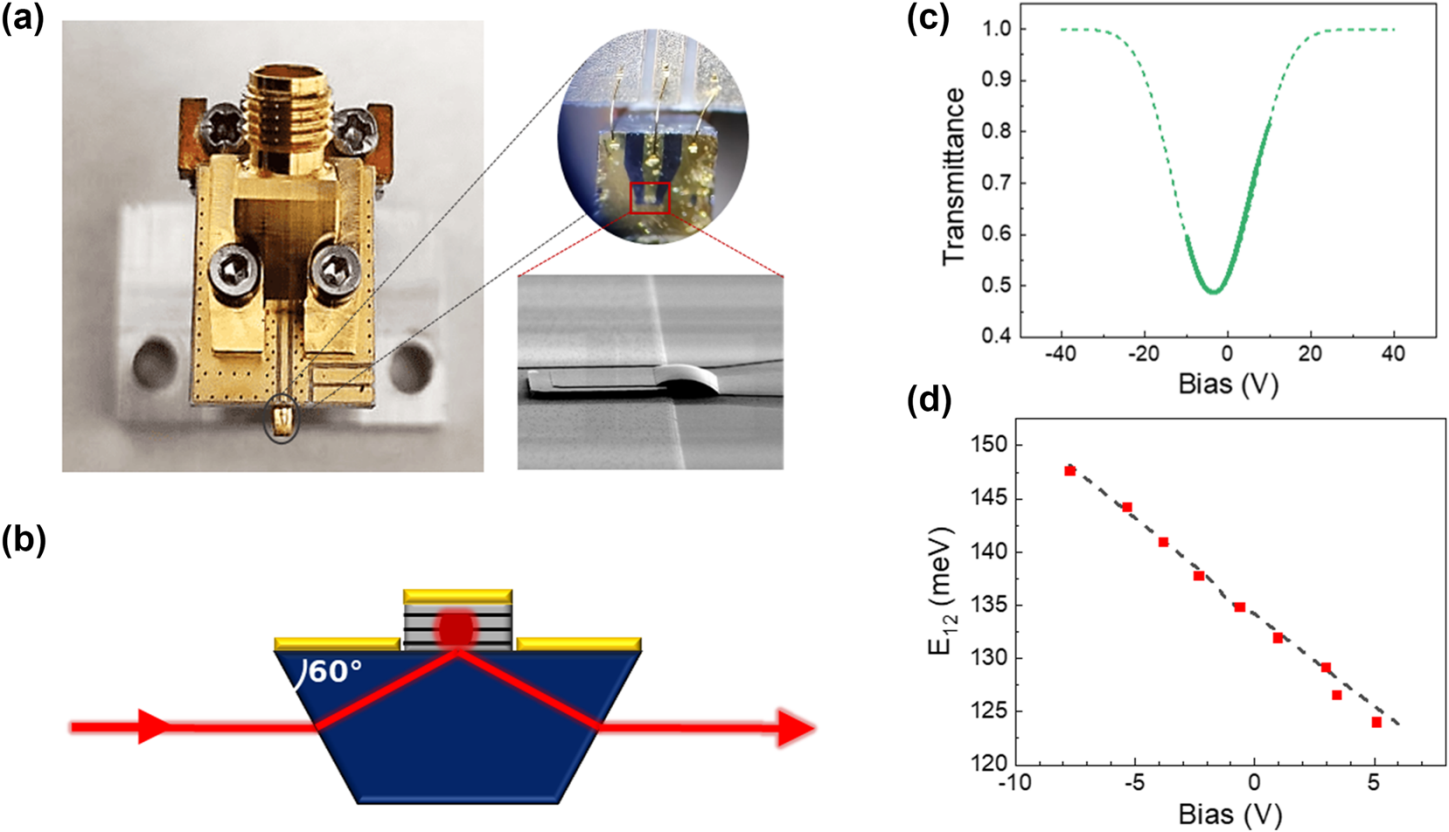
Characteristics of the Stark modulator. (a) Image of the RF packaging of the Stark modulator. The zoom on top is an optical microscope image of the wire bonding between device and PCB, while the bottom zoom is a scanning electron microscope image of the mesa and of the air bridge connecting the mesa with the coplanar waveguide. (b) Sketch of the section of the polished mesa with 60° facets for transmission operation. (c) Simulated (dashed line) and measured (solid line) transmittance of the Stark modulator as a function of the applied bias. (d) Simulated (dashed line) and measured (solid squares) Stark shift.

Our modulator was fabricated in mesa geometry with a high frequency packaging by connecting the mesa to a coplanar waveguide through an air-bridge (see Figure 2a). Light coupling was ensured by polishing the substrate into a prism (see Figure 2b). This configuration allows complying with the polarization selection rule of intraband transitions and facilitates the alignment of the device, as the incident and the transmitted beam are on the same optical axis.

Figure 2c presents the measured (continuous line) and simulated (dashed line) transmittance of the device as a function of the applied voltage for a particular impinging wavelength set by the distributed feedback QCL (9 µm). We find an excellent agreement between measured and simulated transmittance. The modulation depth extracted from the experimental data is 38 % in the voltage range comprised between −10 V and 10 V. It is important to note that the transmittance depends linearly on the voltage in the range between 1 V and 8 V. This is the region where the modulator is operated for the LiDAR–radar demonstration. In this region, the phase modulation is almost vanishing, as it can be seen in the supplementary material.

The Stark shift is determined by measuring the voltage dependent transmittance of the device for different wavelengths. For this measurement, the device is illuminated with a monochromatic beam of a tunable quantum cascade laser (MIRcat-QT Mid-IR Laser) and the transmitted beam is focused on a mercury cadmium telluride detector. Figure 2d reports, for each laser energy, the bias at which the transmittance has its minimum value. This corresponds to the resonance condition between the laser line and the intraband transition energy *E*_12_. From this measurement, a Stark shift of 1.8 meV/V is extracted. These experimental results are in excellent agreement with the calculated intraband transition energy as a function of the voltage, reported as a dashed line in Figure 2d.

**Figure 3:**
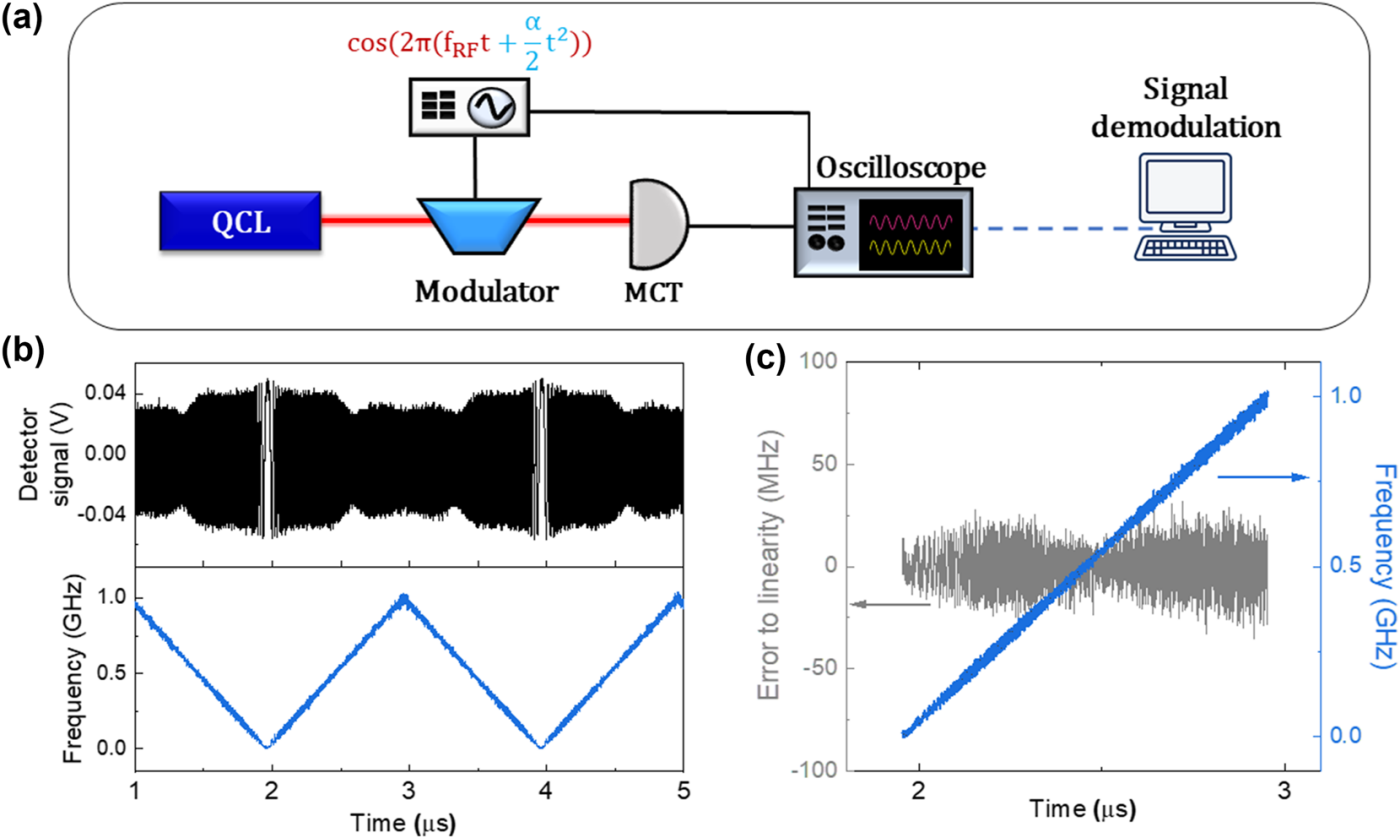
Characterization of the Stark modulator FM linearity. (a) Experimental setup for testing the frequency modulation linearity. (b) Oscilloscope trace of the detector signal (black) and corresponding instantaneous frequency (blue). (c) Estimation of the frequency linearity: the grey plot is the difference between the instantaneous frequency (blue line) and its linear fit, yielding a relative error to linearity of 0.4 %.

In order to use the Stark modulator in our hybrid LiDAR – radar system, it is important to verify its linearity when the modulation frequency is chirped. The experimental set-up is sketched in Figure 3a. The mid-infrared carrier at 9 µm wavelength is transmitted through the modulator and detected by a 1.2 GHz bandwidth mercury cadmium telluride (MCT) detector (VIGO UHSM-10.6) connected to a fast oscilloscope. The bias applied to the modulator is a sinusoidal waveform, produced by an arbitrary waveform generator (AWG Keysight M8195), whose frequency is linearly chirped in a triangular shape from 0 to 1 GHz every µs. The measured photocurrent signal is reported in the top panel of Figure 3b: its Hilbert transform provides the instantaneous phase, whose time derivative is the instantaneous frequency (bottom panel of Figure 3b). Figure 3c presents the error to the linearity (grey line), obtained by calculating the difference between the instantaneous frequency (blue line) and its linear fit. The standard deviation of this error, normalized by the total frequency excursion of 1 GHz, is 0.4 %. These results show that a very good linearity is maintained throughout the entire frequency sweep, without performing any correction. The modulation span *B* = 1 GHz sets the spatial resolution of the distance measurement $\delta d=\frac{c}{2B}=15$ cm. Note that this value is limited in this experiment by the detector’s bandwidth, rather than by the modulator’s one (5 GHz).

## 4 Measurement of the distance and speed

In our hybrid LiDAR–radar system, we use a metamaterial QWIP, fabricated by MOCVD, centered at 9 µm, as coherent receiver. It displays responsivity of 1 A/W at room temperature and frequency bandwidth of 2.3 GHz. Such detector is based on the same design as the one employed in the experiment reported in reference [6] and its full characterization will be presented elsewhere. We apply to the modulator a sinusoidal bias whose frequency is linearly chirped from 100 MHz to 1.1 GHz with a ramp duration T = 1 µs, resulting in a modulation rate *α* = 10^15^ Hz/s.

**Figure 4:**
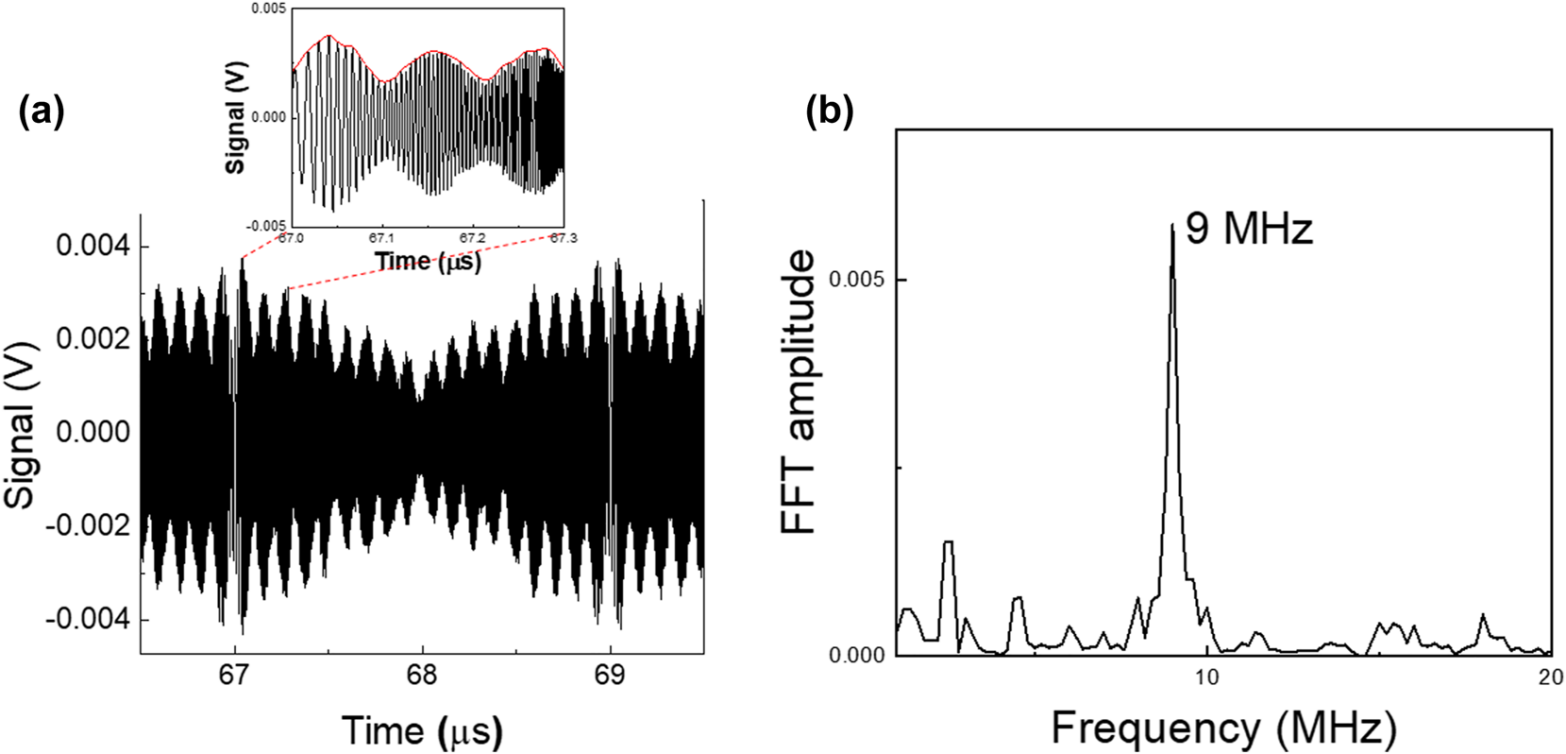
Distance measurement of a still target. (a) Oscilloscope trace with zoom on the envelope (red line). (b) Fast Fourier transform of the envelope for the extraction of the target distance.

We first consider a still target at distance *d* = 1.34 m from the reference mirror. Figure 4a presents the signal on the QWIP resulting from the mixing between ε_ref_ and ε_*S*_. The zoom highlights in red the envelope of the beating signal, whose fast Fourier transform is reported in Figure 4b. From this Fourier transform, we extract the beatnote frequency Δ*f* = 9 MHz, allowing us to deduce the distance, *d* = 1.35 m. The accuracy on the distance measurement is 5 cm, as estimated from the linewidth of the Fourier transform of the beating signal. Note that this quantity does not depend on the linewidth of the QCL, because in our experiment, based on self-homodyne detection, the phase noise of the laser cancels out in the beating.

**Figure 5:**
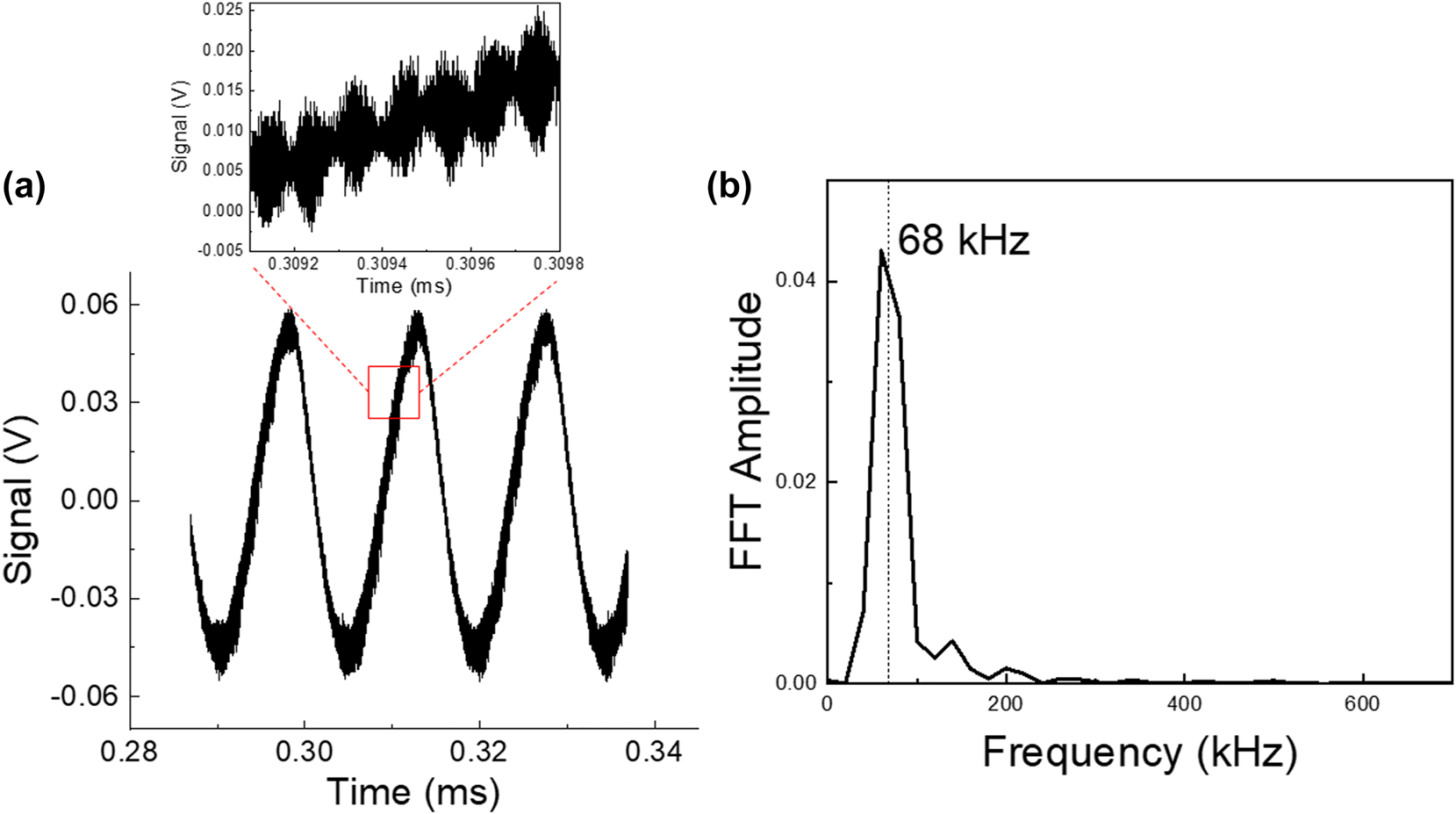
Speed measurement. (a) Oscilloscope waveform; the zoom highlights the heterodyne beatnote. (b) Fast Fourier transform of the temporal trace. The highlighted frequency allows us to determine the speed of the mirror.

The experiment is then repeated while the target mirror is moving back and forth on the delay line, with a speed *v* = 300 mm/s. The signal measured on the coherent receiver is displayed in Figure 5a. Panel (b) presents its Fourier transform, with a peak at 68 kHz, corresponding to the Doppler shift of the mid-infrared carrier. From the value of the Doppler shift we extract the value of the velocity of the target, *v* = 309 mm/s, with an accuracy of 9 mm/s. Note that in a standard FMCW Lidar, the resolution on the speed measurement is $\delta v=\frac{\lambda }{2T}$ , which in our experiment would correspond to *δv* = 4.5 m/s, 15 times larger than the speed of our target. The reason why we are able to measure *v* in our experiment is inherent to the principle of operation of our LiDAR–radar. Indeed, the velocity is directly extracted from the self-homodyne beating between the reference signal and the reflected one. The speed resolution is related to the length of the temporal trace, shown in Figure 5a, or in other words to the integration time T_*meas*_ = 50 µs. The resulting resolution is $\frac{\lambda }{2{\text{T}}_{\text{meas}}}=90\;\text{mm/s}$ . As in the distance measurement, the resolution does not depend on the linewidth of the laser because of our self-homodyne technique.

Note that the signal in Figure 5a also contains information about the measurement of the distance. Indeed, a zoom on the temporal trace, as presented in the inset of Figure 5a, displays several periods of the subcarrier modulation. By performing the Fourier transform of the envelope, the distance measurement is recovered (*d* = 1.5 m in the present experiment).

## 5 Conclusions and perspectives

In this work, we provide a proof of principle of a hybrid LiDAR–radar based on coherent detection, involving a mid-infrared carrier for the measurement of the speed and a radiofrequency subcarrier for the measurement of the distance. Note that the measurements of velocity and distance are made in parallel and independently.

Our system is entirely based on unipolar quantum optoelectronic devices, which enable to transfer RF electronics functions into the optical domain, thus giving rise to optical microwave systems in the mid infrared wavelength region. The quantum cascade laser provides a directional and powerful carrier at 9 µm, in one of the transparency windows of the atmosphere. The Stark modulator is used to create an RF subcarrier, which is chirped employing standard radar waveform generators. The modulator ensures a linear response to the frequency chirp that can be used for the measurement of the distance without the need for slope predistortion corrections. Finally, the metamaterial quantum well infrared photodetector is an excellent coherent receiver, thanks to its large frequency bandwidth and high saturation power. All the devices operate at room temperature. Our work thus demonstrates that unipolar quantum optoelectronics can become an interesting player among photonic systems for detection and ranging [23].

Several improvements can be envisioned. Metamaterials can provide an important increase of the bandwidth of unipolar detectors [13] and modulators [5] and therefore will contribute to substantially increase the resolution. Furthermore, the realisation of phase [14] rather than amplitude modulators could foster the development of FMCW LiDARs, based on the phase modulation of the carrier.

## Supplementary Material

Supplementary Material Details
