# Supplementary material for: Hybrid LiDAR–radar at 9 μm wavelength with unipolar quantum optoelectronic devices
Source: Nanophotonics. 2025 Sep 1;14(21):3407–13. doi: 10.1515/nanoph-2025-0265 (PMC12552881; doi:10.1515/nanoph-2025-0265)
Supplement: Supplementary file 1 — Supplementary Material Details [file j_nanoph-2025-0265_suppl_001.pdf]

Hybrid Lidar-Radar at 9µm wavelength with unipolar quantum optoelectronic devices

Supporting information

Livia Del Balzo[1], Djamal Gacemi[1], Jihye Baik[1], Bruno Martin[1,2], Axel Evirgen[3], Grégoire Beaudoin[4], Konstantinos Pantzas[4], Isabelle Sagnes[4], Angela Vasanelli[5], Carlo Sirtori[1]

1. Laboratoire de Physique de l'École normale supérieure, ENS, Université PSL, CNRS, Sorbonne Université, Université Paris Cité, 75005 Paris, France
2. Thales Research and Technology, Palaiseau, France
3. III-V Lab, Palaiseau, 91120 Palaiseau, France
4. Centre de Nanosciences et de Nanotechnologies, Université Paris-Saclay, CNRS, Palaiseau, 91120, France
5. Laboratoire de Physique de l'ENS, Université Paris Cité, Ecole normale supérieure, Université PSL, Sorbonne Université, CNRS, 75005 Paris, France

1. Stark modulator phase properties

In this paragraph, we present the experimental characterization of the phase modulation occurring simultaneously to the amplitude modulation in the device, as described by the Kramers-Kronig relations. We exploit a stabilized heterodyne setup similar to that of ref. [1] using two distributed feedback (DFB) quantum cascade lasers (QCLs) at 9µm, as sketched in Figure S.1.

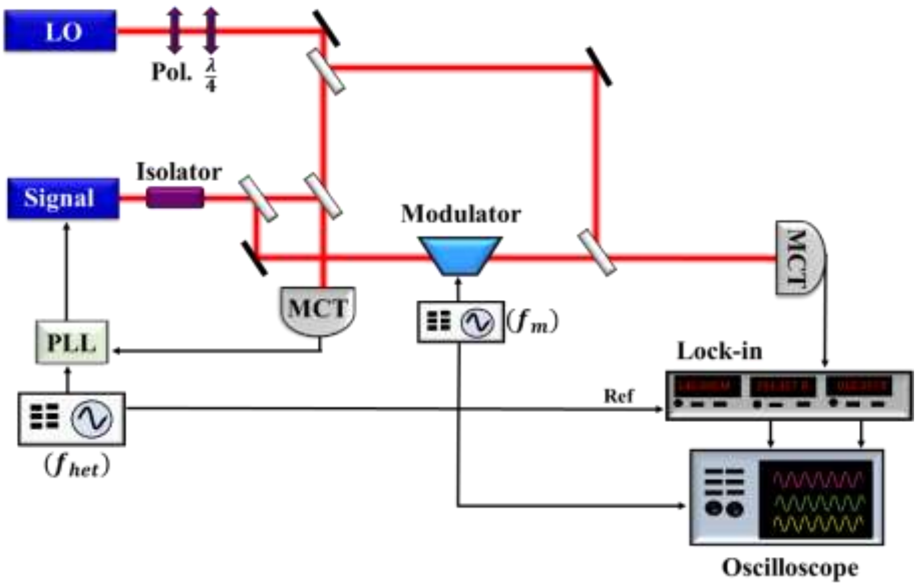

Figure S.1. Experimental setup for the phase modulation measurement. An optical isolator and a combination of polarizer (Pol.) and quarter waveplate (λ /4) are used to avoid feedback in the QCLs. Two commercial mercury cadmium telluride (MCT) detectors are used for the stabilization and measurement arm. A signal generator at f_{het}= 140 MHz is the reference for the phase – locked loop (PLL) and the lock-in, while another signal at f_m= 10 kHz is applied to the modulator, which is directly observable on the oscilloscope.

The stabilization arm is essential since the goal is to extract the phase introduced by the modulator and it is thus important to get rid of any other phase fluctuation, in particular the one due to the QCLs. The PLL ensures that the phase difference between the two lasers remains constant and allows us to isolate the modulator's

contribution. On the second arm, another MCT device detects the beatnote between the modulated signal laser (both in amplitude and phase) and the local oscillator (LO) laser.

An optical isolator is placed after the signal laser to prevent feedback effects (i.e. the light reflected by the modulator's facet is re-injected in the QCL cavity). A polarizer and a quarter wave-plate are used after the local oscillator to both prevent residual feedback and attenuate the optical power to avoid saturation of the MCT detector. A lock-in amplifier (UHFLI, Zürich Instrument), whose reference is the same as the one of the phase-locked loop (PLL) (f_{het}= 140 MHz), extracts the amplitude and phase of the heterodyne signal. The lock-in bandwidth around the reference frequency can be adapted to include the modulation frequency of the applied bias, so that it is possible to follow the time evolution of the bias and of the phase. For an applied modulation frequency f_m=10 kHz, a bandwidth of 30 kHz is set.

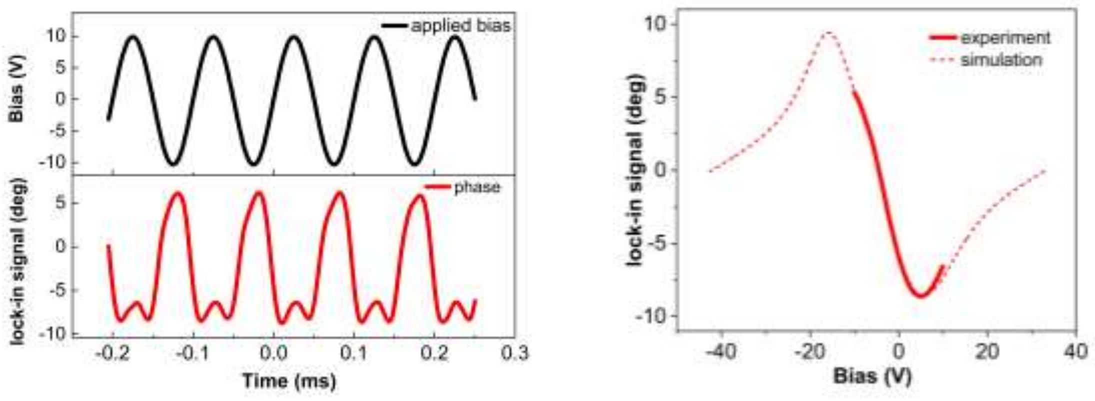

a) b)

Figure S.2 Phase modulation measurement. (a) Applied voltage and phase visualized on the oscilloscope. (b) In solid line, plot of the phase as a function of the applied voltage. In dashed lines, the simulation performed by applying the Hilbert transform to the device absorption spectrum.

Figure S.2 presents the lock-in phase. Its time evolution together with that of the applied bias is shown in panel (a), while panel (b) displays the phase variation with bias. The experimental data (solid lines), averaged over 1000 acquisitions to reduce the noise, are in good agreement with the phase calculated as the Hilbert transform of the amplitude modulation (dashed lines). As we can see, in the range [−10V, 10V] a phase modulation of 14 degrees is obtained. In the lidar-radar experiment, the modulator operates within the linear absorption region, ranging approximately from 1 V to 8 V, which coincides with a minimum in the phase modulation response. This confirms that the optical carrier phase is barely affected within this operating range.

References

1. Dely, H. *et al.* Heterodyne coherent detection of phase modulation in a mid-infrared unipolar device. *Opt. Express* **31**, 30876 (2023).
